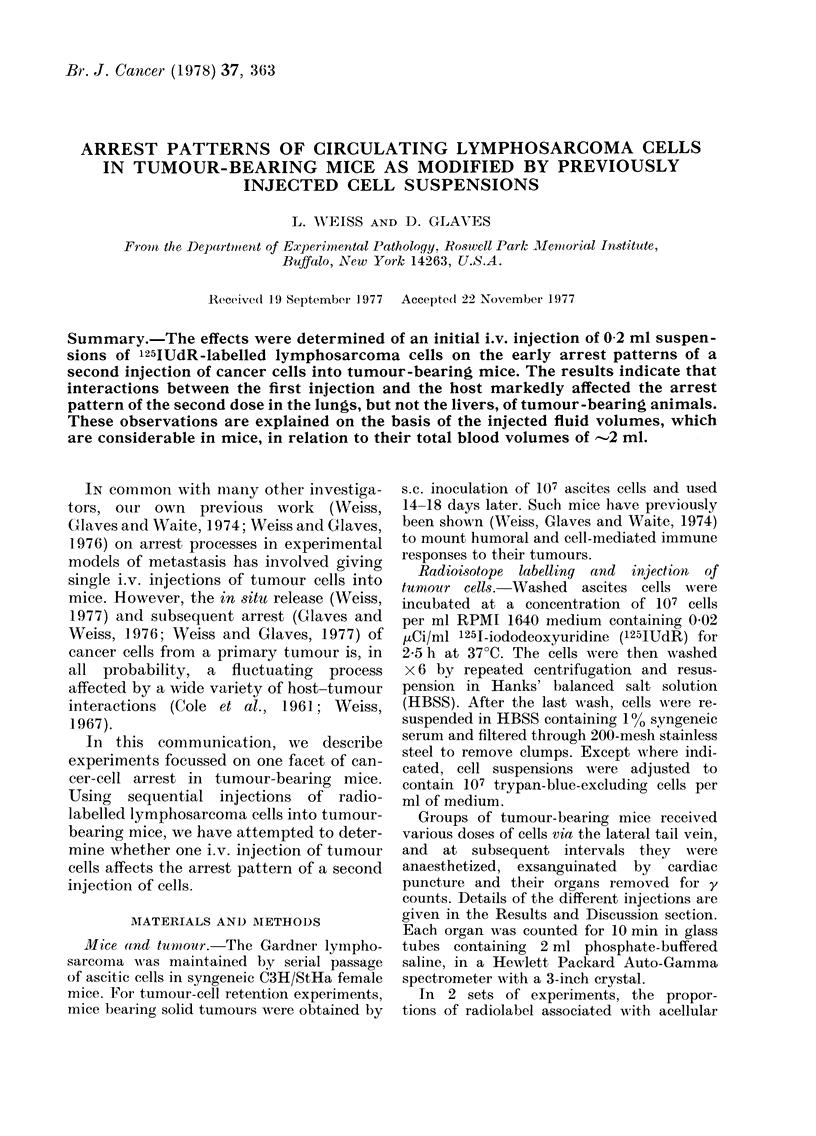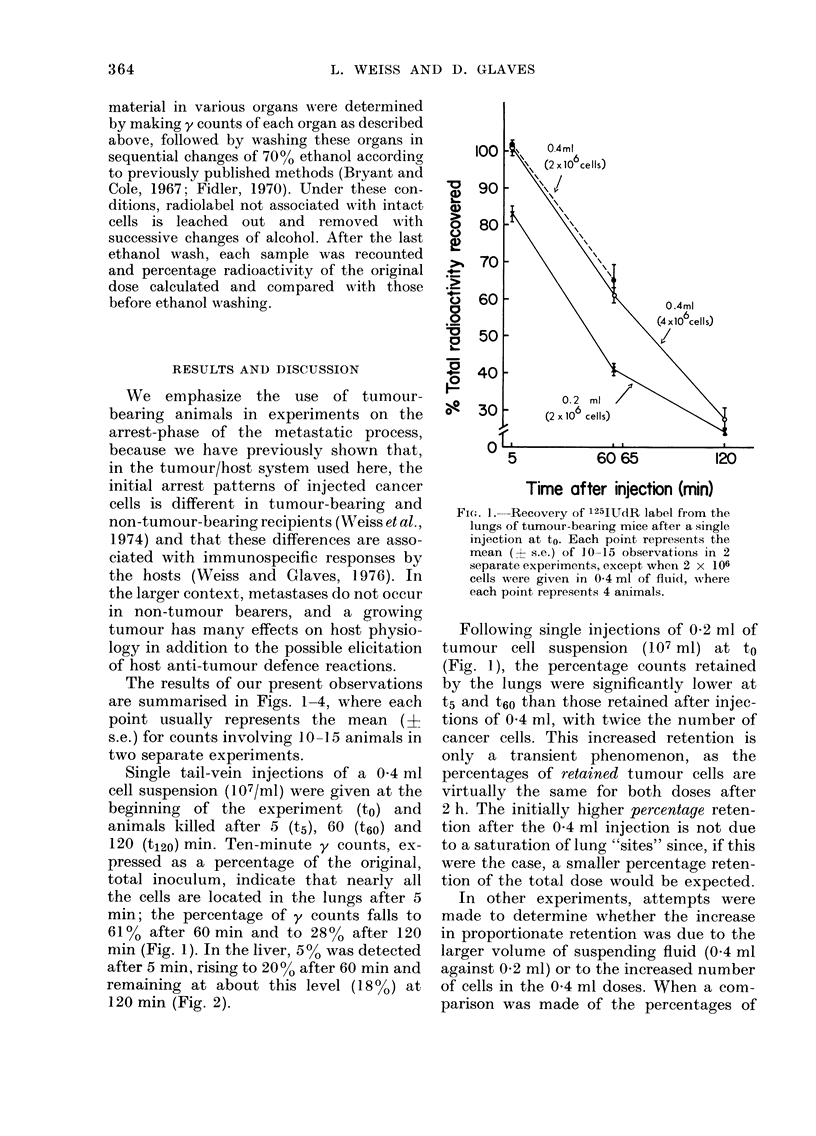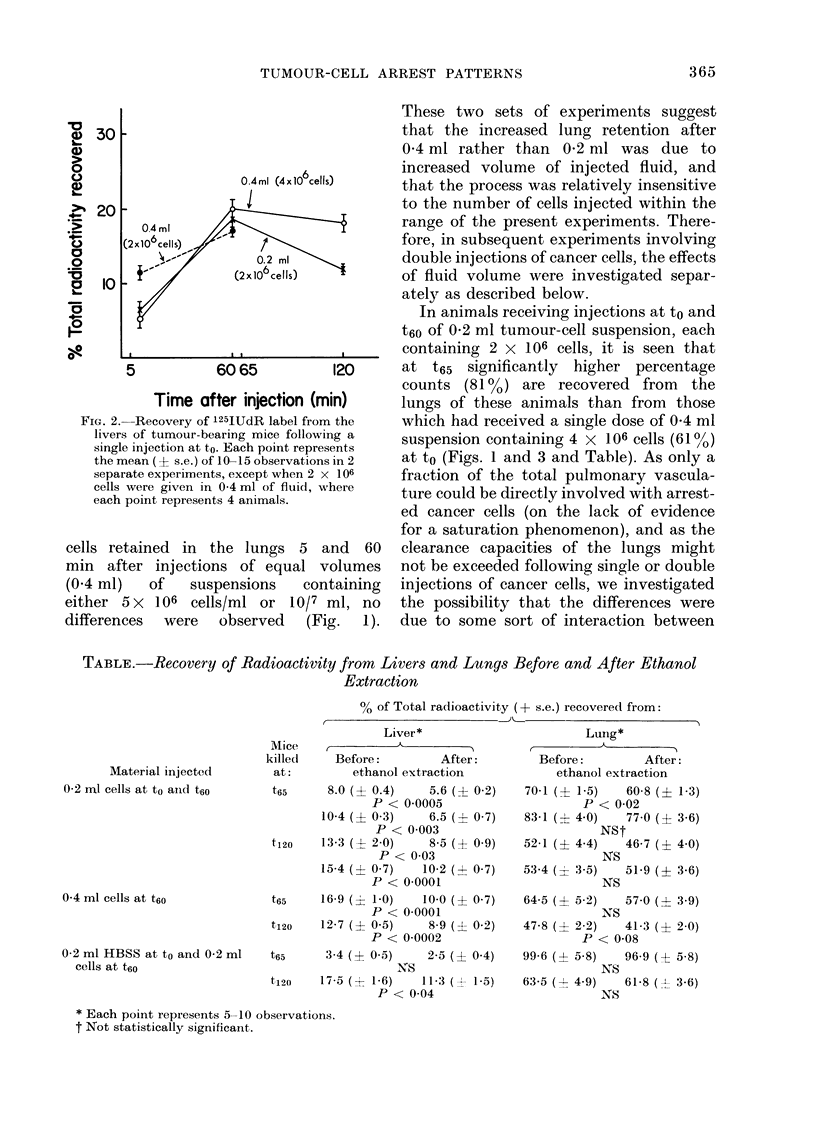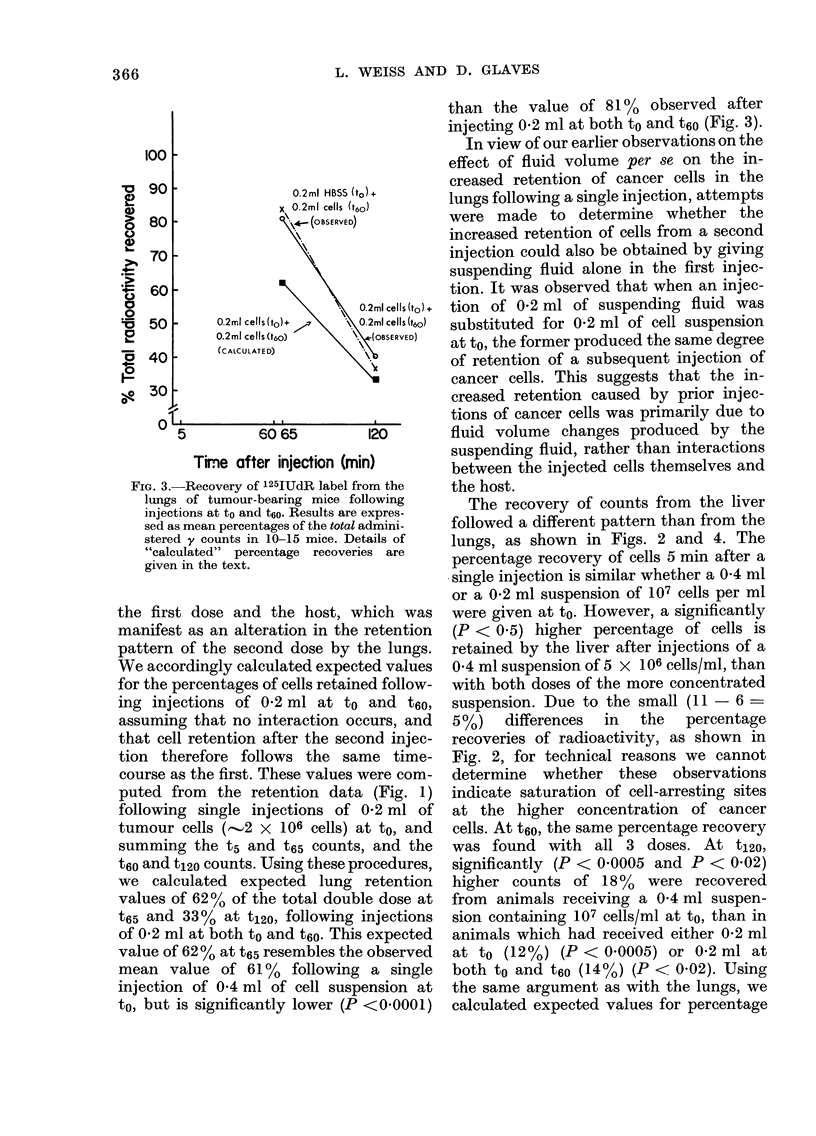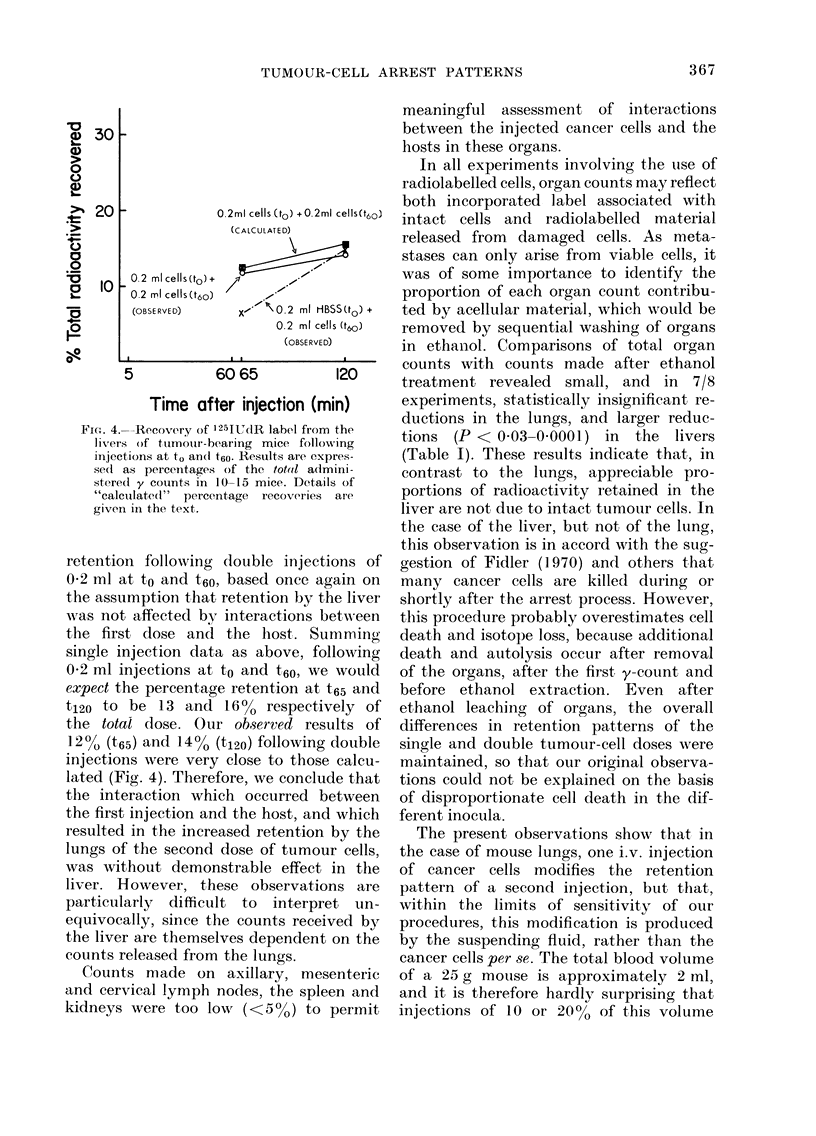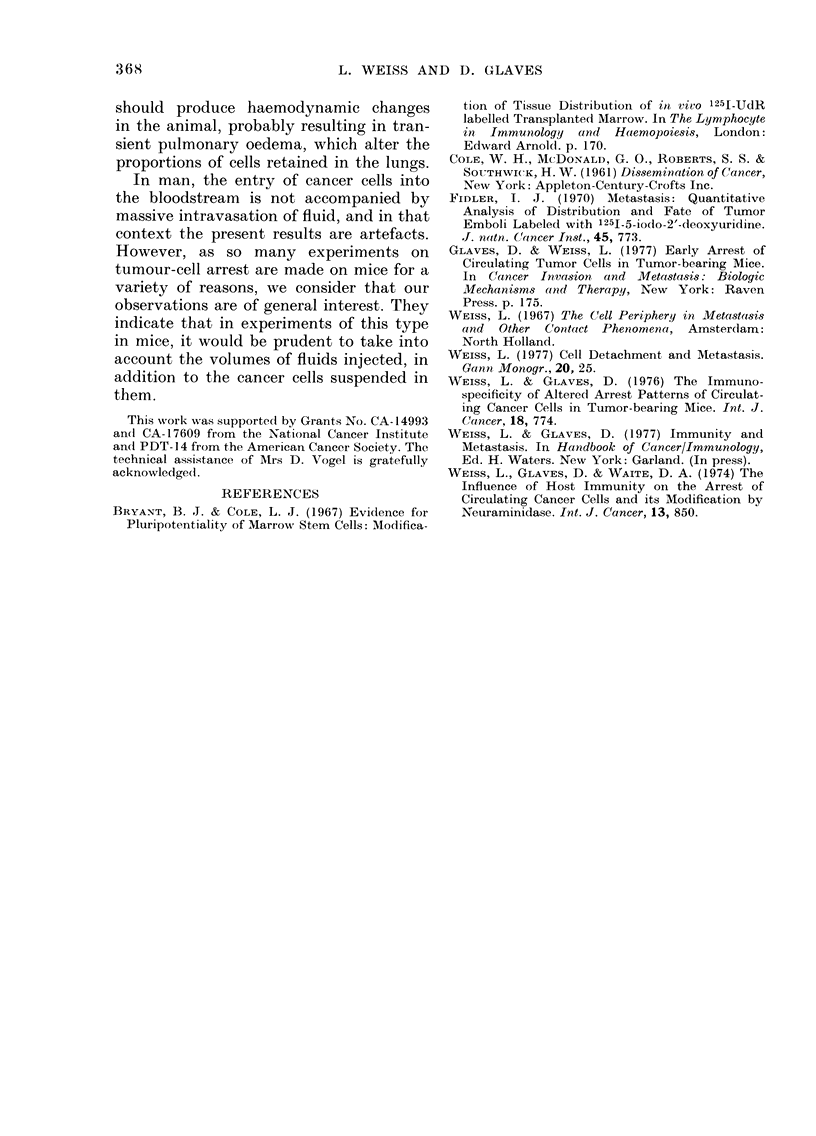# Arrest patterns of circulating lymphosarcoma cells in tumour-bearing mice as modified by previously injected cell suspensions.

**DOI:** 10.1038/bjc.1978.55

**Published:** 1978-03

**Authors:** L. Weiss, D. Glaves

## Abstract

The effects were determined of an initial i.v. injection of 0.2 ml suspensions of 125IUdR-labelled lymphosarcoma cells on the early arrest patterns of a second injection of cancer cells into tumour-bearing mice. The results indicate that interactions between the first injection and the host markedly affected the arrest pattern of the second dose in the lungs, but not the livers, of tumour-bearing animals. These observations are explained on the basis of the injected fluid volumes, which are considerable in mice, in relation to their total blood volumes of approximately 2 ml.


Br. J. Cancer (1978) 37, 363

### ARREST PATTERNS OF CIRCULATING LYMPHOSARCOMA CELLS IN TUMOUR-BEARING MICE AS MODIFIED BY PREVIOUSLY INJECTED CELL SUSPENSIONS

L. WEISS AND D. GLAVES

*From the Department of Experimental Pathology, Roswell Park Memorial Institute, Buffalo, New York* 14263, *U.S.A.*

Received 19 September 1977 Accepted 22 November 1977

**Summary.—The effects were determined of an initial i.v. injection of 0·2 ml suspensions of $^{125}$IUdR-labelled lymphosarcoma cells on the early arrest patterns of a second injection of cancer cells into tumour-bearing mice. The results indicate that interactions between the first injection and the host markedly affected the arrest pattern of the second dose in the lungs, but not the livers, of tumour-bearing animals. These observations are explained on the basis of the injected fluid volumes, which are considerable in mice, in relation to their total blood volumes of ~2 ml.**

In common with many other investigators, our own previous work (Weiss, Glaves and Waite, 1974; Weiss and Glaves, 1976) on arrest processes in experimental models of metastasis has involved giving single i.v. injections of tumour cells into mice. However, the *in situ* release (Weiss, 1977) and subsequent arrest (Glaves and Weiss, 1976; Weiss and Glaves, 1977) of cancer cells from a primary tumour is, in all probability, a fluctuating process affected by a wide variety of host–tumour interactions (Cole *et al.*, 1961; Weiss, 1967).

In this communication, we describe experiments focussed on one facet of cancer-cell arrest in tumour-bearing mice. Using sequential injections of radiolabelled lymphosarcoma cells into tumour-bearing mice, we have attempted to determine whether one i.v. injection of tumour cells affects the arrest pattern of a second injection of cells.

### MATERIALS AND METHODS

*Mice and tumour.*—The Gardner lymphosarcoma was maintained by serial passage of ascitic cells in syngeneic C3H/StHa female mice. For tumour-cell retention experiments, mice bearing solid tumours were obtained by s.c. inoculation of $10^7$ ascites cells and used 14–18 days later. Such mice have previously been shown (Weiss, Glaves and Waite, 1974) to mount humoral and cell-mediated immune responses to their tumours.

*Radioisotope labelling and injection of tumour cells.*—Washed ascites cells were incubated at a concentration of $10^7$ cells per ml RPMI 1640 medium containing 0·02 $\mu$Ci/ml $^{125}$I-iododeoxyuridine ($^{125}$IUdR) for 2·5 h at 37°C. The cells were then washed ×6 by repeated centrifugation and resuspension in Hanks' balanced salt solution (HBSS). After the last wash, cells were resuspended in HBSS containing 1% syngeneic serum and filtered through 200-mesh stainless steel to remove clumps. Except where indicated, cell suspensions were adjusted to contain $10^7$ trypan-blue-excluding cells per ml of medium.

Groups of tumour-bearing mice received various doses of cells *via* the lateral tail vein, and at subsequent intervals they were anaesthetized, exsanguinated by cardiac puncture and their organs removed for $\gamma$ counts. Details of the different injections are given in the Results and Discussion section. Each organ was counted for 10 min in glass tubes containing 2 ml phosphate-buffered saline, in a Hewlett Packard Auto-Gamma spectrometer with a 3-inch crystal.

In 2 sets of experiments, the proportions of radiolabel associated with acellular material in various organs were determined by making $\gamma$ counts of each organ as described above, followed by washing these organs in sequential changes of 70% ethanol according to previously published methods (Bryant and Cole, 1967; Fidler, 1970). Under these conditions, radiolabel not associated with intact cells is leached out and removed with successive changes of alcohol. After the last ethanol wash, each sample was recounted and percentage radioactivity of the original dose calculated and compared with those before ethanol washing.

### RESULTS AND DISCUSSION

We emphasize the use of tumour-bearing animals in experiments on the arrest-phase of the metastatic process, because we have previously shown that, in the tumour/host system used here, the initial arrest patterns of injected cancer cells is different in tumour-bearing and non-tumour-bearing recipients (Weiss *et al.*, 1974) and that these differences are associated with immunospecific responses by the hosts (Weiss and Glaves, 1976). In the larger context, metastases do not occur in non-tumour bearers, and a growing tumour has many effects on host physiology in addition to the possible elicitation of host anti-tumour defence reactions.

The results of our present observations are summarised in Figs. 1–4, where each point usually represents the mean ($\pm$ s.e.) for counts involving 10–15 animals in two separate experiments.

Single tail-vein injections of a 0·4 ml cell suspension ($10^7$/ml) were given at the beginning of the experiment ($t_0$) and animals killed after 5 ($t_5$), 60 ($t_{60}$) and 120 ($t_{120}$) min. Ten-minute $\gamma$ counts, expressed as a percentage of the original, total inoculum, indicate that nearly all the cells are located in the lungs after 5 min; the percentage of $\gamma$ counts falls to 61% after 60 min and to 28% after 120 min (Fig. 1). In the liver, 5% was detected after 5 min, rising to 20% after 60 min and remaining at about this level (18%) at 120 min (Fig. 2).

FIG. 1.—Recovery of $^{125}$IUdR label from the lungs of tumour-bearing mice after a single injection at $t_0$. Each point represents the mean ($\pm$ s.e.) of 10–15 observations in 2 separate experiments, except when 2 $\times$ $10^6$ cells were given in 0·4 ml of fluid, where each point represents 4 animals.

Following single injections of 0·2 ml of tumour cell suspension ($10^7$ ml) at $t_0$ (Fig. 1), the percentage counts retained by the lungs were significantly lower at $t_5$ and $t_{60}$ than those retained after injections of 0·4 ml, with twice the number of cancer cells. This increased retention is only a transient phenomenon, as the percentages of *retained* tumour cells are virtually the same for both doses after 2 h. The initially higher *percentage* retention after the 0·4 ml injection is not due to a saturation of lung "sites" since, if this were the case, a smaller percentage retention of the total dose would be expected.

In other experiments, attempts were made to determine whether the increase in proportionate retention was due to the larger volume of suspending fluid (0·4 ml against 0·2 ml) or to the increased number of cells in the 0·4 ml doses. When a comparison was made of the percentages of

FIG. 2.—Recovery of $^{125}$IUdR label from the livers of tumour-bearing mice following a single injection at $t_0$. Each point represents the mean (± s.e.) of 10–15 observations in 2 separate experiments, except when 2 × $10^6$ cells were given in 0·4 ml of fluid, where each point represents 4 animals.

cells retained in the lungs 5 and 60 min after injections of equal volumes (0·4 ml) of suspensions containing either 5× $10^6$ cells/ml or $10/^7$ ml, no differences were observed (Fig. 1). These two sets of experiments suggest that the increased lung retention after 0·4 ml rather than 0·2 ml was due to increased volume of injected fluid, and that the process was relatively insensitive to the number of cells injected within the range of the present experiments. Therefore, in subsequent experiments involving double injections of cancer cells, the effects of fluid volume were investigated separately as described below.

In animals receiving injections at $t_0$ and $t_{60}$ of 0·2 ml tumour-cell suspension, each containing 2 × $10^6$ cells, it is seen that at $t_{65}$ significantly higher percentage counts (81%) are recovered from the lungs of these animals than from those which had received a single dose of 0·4 ml suspension containing 4 × $10^6$ cells (61%) at $t_0$ (Figs. 1 and 3 and Table). As only a fraction of the total pulmonary vasculature could be directly involved with arrested cancer cells (on the lack of evidence for a saturation phenomenon), and as the clearance capacities of the lungs might not be exceeded following single or double injections of cancer cells, we investigated the possibility that the differences were due to some sort of interaction between

TABLE.—*Recovery of Radioactivity from Livers and Lungs Before and After Ethanol Extraction*

| | | % of Total radioactivity (+ s.e.) recovered from: | | | |
|---|---|---|---|---|---|
| | | Liver* | | Lung* | |
| Material injected | Mice killed at: | Before: ethanol extraction | After: ethanol extraction | Before: ethanol extraction | After: ethanol extraction |
| 0·2 ml cells at $t_0$ and $t_{60}$ | $t_{65}$ | 8.0 (± 0.4) | 5.6 (± 0·2) | 70·1 (± 1·5) | 60·8 (± 1·3) |
| | | $P < 0·0005$ | | $P < 0·02$ | |
| | | 10·4 (± 0·3) | 6.5 (± 0·7) | 83·1 (± 4·0) | 77·0 (± 3·6) |
| | | $P < 0·003$ | | NS† | |
| | $t_{120}$ | 13·3 (± 2·0) | 8·5 (± 0·9) | 52·1 (± 4·4) | 46·7 (± 4·0) |
| | | $P < 0·03$ | | NS | |
| | | 15·4 (± 0·7) | 10·2 (± 0·7) | 53·4 (± 3·5) | 51·9 (± 3·6) |
| | | $P < 0·0001$ | | NS | |
| 0·4 ml cells at $t_{60}$ | $t_{65}$ | 16·9 (± 1·0) | 10·0 (± 0·7) | 64·5 (± 5·2) | 57·0 (± 3·9) |
| | | $P < 0·0001$ | | NS | |
| | $t_{120}$ | 12·7 (± 0·5) | 8·9 (± 0·2) | 47·8 (± 2·2) | 41·3 (± 2·0) |
| | | $P < 0·0002$ | | $P < 0·08$ | |
| 0·2 ml HBSS at $t_0$ and 0·2 ml cells at $t_{60}$ | $t_{65}$ | 3·4 (± 0·5) | 2·5 (± 0·4) | 99·6 (± 5·8) | 96·9 (± 5·8) |
| | | NS | | NS | |
| | $t_{120}$ | 17·5 (± 1·6) | 11·3 (± 1·5) | 63·5 (± 4·9) | 61·8 (± 3·6) |
| | | $P < 0·04$ | | NS | |

* Each point represents 5–10 observations.
† Not statistically significant.

FIG. 3.—Recovery of $^{125}$IUdR label from the lungs of tumour-bearing mice following injections at $t_0$ and $t_{60}$. Results are expressed as mean percentages of the *total* administered $\gamma$ counts in 10–15 mice. Details of "calculated" percentage recoveries are given in the text.

the first dose and the host, which was manifest as an alteration in the retention pattern of the second dose by the lungs. We accordingly calculated expected values for the percentages of cells retained following injections of 0·2 ml at $t_0$ and $t_{60}$, assuming that no interaction occurs, and that cell retention after the second injection therefore follows the same time-course as the first. These values were computed from the retention data (Fig. 1) following single injections of 0·2 ml of tumour cells ($\sim 2 \times 10^6$ cells) at $t_0$, and summing the $t_5$ and $t_{65}$ counts, and the $t_{60}$ and $t_{120}$ counts. Using these procedures, we calculated expected lung retention values of 62% of the total double dose at $t_{65}$ and 33% at $t_{120}$, following injections of 0·2 ml at both $t_0$ and $t_{60}$. This expected value of 62% at $t_{65}$ resembles the observed mean value of 61% following a single injection of 0·4 ml of cell suspension at $t_0$, but is significantly lower ($P < 0{\cdot}0001$) than the value of 81% observed after injecting 0·2 ml at both $t_0$ and $t_{60}$ (Fig. 3).

In view of our earlier observations on the effect of fluid volume *per se* on the increased retention of cancer cells in the lungs following a single injection, attempts were made to determine whether the increased retention of cells from a second injection could also be obtained by giving suspending fluid alone in the first injection. It was observed that when an injection of 0·2 ml of suspending fluid was substituted for 0·2 ml of cell suspension at $t_0$, the former produced the same degree of retention of a subsequent injection of cancer cells. This suggests that the increased retention caused by prior injections of cancer cells was primarily due to fluid volume changes produced by the suspending fluid, rather than interactions between the injected cells themselves and the host.

The recovery of counts from the liver followed a different pattern than from the lungs, as shown in Figs. 2 and 4. The percentage recovery of cells 5 min after a single injection is similar whether a 0·4 ml or a 0·2 ml suspension of $10^7$ cells per ml were given at $t_0$. However, a significantly ($P < 0{\cdot}5$) higher percentage of cells is retained by the liver after injections of a 0·4 ml suspension of $5 \times 10^6$ cells/ml, than with both doses of the more concentrated suspension. Due to the small ($11 - 6 = 5\%$) differences in the percentage recoveries of radioactivity, as shown in Fig. 2, for technical reasons we cannot determine whether these observations indicate saturation of cell-arresting sites at the higher concentration of cancer cells. At $t_{60}$, the same percentage recovery was found with all 3 doses. At $t_{120}$, significantly ($P < 0{\cdot}0005$ and $P < 0{\cdot}02$) higher counts of 18% were recovered from animals receiving a 0·4 ml suspension containing $10^7$ cells/ml at $t_0$, than in animals which had received either 0·2 ml at $t_0$ (12%) ($P < 0{\cdot}0005$) or 0·2 ml at both $t_0$ and $t_{60}$ (14%) ($P < 0{\cdot}02$). Using the same argument as with the lungs, we calculated expected values for percentage

FIG. 4.—Recovery of $^{125}$IUdR label from the livers of tumour-bearing mice following injections at $t_0$ and $t_{60}$. Results are expressed as percentages of the *total* administered $\gamma$ counts in 10–15 mice. Details of "calculated" percentage recoveries are given in the text.

retention following double injections of 0·2 ml at $t_0$ and $t_{60}$, based once again on the assumption that retention by the liver was not affected by interactions between the first dose and the host. Summing single injection data as above, following 0·2 ml injections at $t_0$ and $t_{60}$, we would *expect* the percentage retention at $t_{65}$ and $t_{120}$ to be 13 and 16% respectively of the *total* dose. Our *observed* results of 12% ($t_{65}$) and 14% ($t_{120}$) following double injections were very close to those calculated (Fig. 4). Therefore, we conclude that the interaction which occurred between the first injection and the host, and which resulted in the increased retention by the lungs of the second dose of tumour cells, was without demonstrable effect in the liver. However, these observations are particularly difficult to interpret unequivocally, since the counts received by the liver are themselves dependent on the counts released from the lungs.

Counts made on axillary, mesenteric and cervical lymph nodes, the spleen and kidneys were too low (<5%) to permit meaningful assessment of interactions between the injected cancer cells and the hosts in these organs.

In all experiments involving the use of radiolabelled cells, organ counts may reflect both incorporated label associated with intact cells and radiolabelled material released from damaged cells. As metastases can only arise from viable cells, it was of some importance to identify the proportion of each organ count contributed by acellular material, which would be removed by sequential washing of organs in ethanol. Comparisons of total organ counts with counts made after ethanol treatment revealed small, and in 7/8 experiments, statistically insignificant reductions in the lungs, and larger reductions ($P < 0{\cdot}03$–$0{\cdot}0001$) in the livers (Table I). These results indicate that, in contrast to the lungs, appreciable proportions of radioactivity retained in the liver are not due to intact tumour cells. In the case of the liver, but not of the lung, this observation is in accord with the suggestion of Fidler (1970) and others that many cancer cells are killed during or shortly after the arrest process. However, this procedure probably overestimates cell death and isotope loss, because additional death and autolysis occur after removal of the organs, after the first $\gamma$-count and before ethanol extraction. Even after ethanol leaching of organs, the overall differences in retention patterns of the single and double tumour-cell doses were maintained, so that our original observations could not be explained on the basis of disproportionate cell death in the different inocula.

The present observations show that in the case of mouse lungs, one i.v. injection of cancer cells modifies the retention pattern of a second injection, but that, within the limits of sensitivity of our procedures, this modification is produced by the suspending fluid, rather than the cancer cells *per se*. The total blood volume of a 25 g mouse is approximately 2 ml, and it is therefore hardly surprising that injections of 10 or 20% of this volume should produce haemodynamic changes in the animal, probably resulting in transient pulmonary oedema, which alter the proportions of cells retained in the lungs.

In man, the entry of cancer cells into the bloodstream is not accompanied by massive intravasation of fluid, and in that context the present results are artefacts. However, as so many experiments on tumour-cell arrest are made on mice for a variety of reasons, we consider that our observations are of general interest. They indicate that in experiments of this type in mice, it would be prudent to take into account the volumes of fluids injected, in addition to the cancer cells suspended in them.

This work was supported by Grants No. CA-14993 and CA-17609 from the National Cancer Institute and PDT-14 from the American Cancer Society. The technical assistance of Mrs D. Vogel is gratefully acknowledged.

### REFERENCES

BRYANT, B. J. & COLE, L. J. (1967) Evidence for Pluripotentiality of Marrow Stem Cells: Modification of Tissue Distribution of *in vivo* $^{125}$I-UdR labelled Transplanted Marrow. In *The Lymphocyte in Immunology and Haemopoiesis*, London: Edward Arnold. p. 170.

COLE, W. H., MCDONALD, G. O., ROBERTS, S. S. & SOUTHWICK, H. W. (1961) *Dissemination of Cancer*, New York: Appleton-Century-Crofts Inc.

FIDLER, I. J. (1970) Metastasis: Quantitative Analysis of Distribution and Fate of Tumor Emboli Labeled with $^{125}$I-5-iodo-2′-deoxyuridine. *J. natn. Cancer Inst.*, **45,** 773.

GLAVES, D. & WEISS, L. (1977) Early Arrest of Circulating Tumor Cells in Tumor-bearing Mice. In *Cancer Invasion and Metastasis: Biologic Mechanisms and Therapy*, New York: Raven Press. p. 175.

WEISS, L. (1967) *The Cell Periphery in Metastasis and Other Contact Phenomena*, Amsterdam: North Holland.

WEISS, L. (1977) Cell Detachment and Metastasis. *Gann Monogr.*, **20,** 25.

WEISS, L. & GLAVES, D. (1976) The Immunospecificity of Altered Arrest Patterns of Circulating Cancer Cells in Tumor-bearing Mice. *Int. J. Cancer*, **18,** 774.

WEISS, L. & GLAVES, D. (1977) Immunity and Metastasis. In *Handbook of Cancer/Immunology*, Ed. H. Waters. New York: Garland. (In press).

WEISS, L., GLAVES, D. & WAITE, D. A. (1974) The Influence of Host Immunity on the Arrest of Circulating Cancer Cells and its Modification by Neuraminidase. *Int. J. Cancer*, **13,** 850.